# An Overview of Microparticulate Drug Delivery System and its Extensive Therapeutic Applications in Diabetes

**DOI:** 10.34172/apb.2022.075

**Published:** 2021-10-04

**Authors:** Mona Hassan Rafiee, Bazigha K. Abdul Rasool

**Affiliations:** Pharmaceutics Department, Dubai Pharmacy College for Girls, Dubai, UAE.

**Keywords:** Microparticles, Antidiabetic drugs, Insulin, Hypoglycemic drugs, Diabetes, Targeting

## Abstract

Microparticulate drug delivery system (MDDS) has attained much consideration in the modern era due to its effectiveness in overcoming traditional treatment problems. Microparticles (MPs) are spherical particles of a diameter ranging from 10 μm to 1000 μm. MPs can encapsulate both water-soluble and insoluble compounds. MDDS proved their efficacy in improving drugs bioavailability, stability, targeting, and controlling their release patterns. MPs also offer comfort, easy administration, and improvement in patient compliance by reducing drugs toxicity and dosage frequency. This review elucidates the fabrication techniques, drug release, and therapeutic application of MDDS. Further details concerning the therapeutic applications of antidiabetic drugs-loaded MPs were also reviewed, including controlling drugs release by gastroretention, improving drugs dissolution, reducing side effects, localizing drugs to the site of disease, improving insulin stability, natural products loaded with MPs, sustained drug release, mucosal delivery, and administration routes. Additionally, the current situation and future prospects in developing MPs loaded with antidiabetic drugs were discussed.

## Introduction

**Figure 1:**
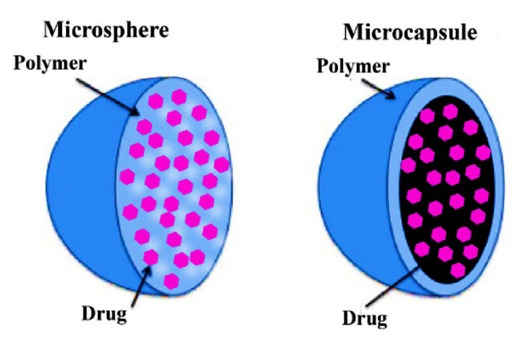
Structure of microparticles: a) matrix microparticle; and b) microcapsule

Microparticles (MPs) are small spherical entities with a diameter ranging from 10 μm to 1000 μm, in the form of free-flowing powders.^1^ They are developed from different components as inorganic, polymeric, and minerals. In addition, MPs can exist in various structural designs, for example, microgranules, micropellets, microcapsules, microsponges, microemulsions, magnetic MPs and lipid vesicles as liposomes and niosomes.^2^ The most common type of MPs are the polymeric MPs, which are made from natural biodegradable or synthetic polymers and designed into two main structures MPs and microspheres. The matrix of the MPs consists of a homogeneous mixture of polymers, copolymer, and active pharmaceutical ingredient (API). Meanwhile, microspheres refer to a core comprised of either solid or liquid surrounded by a coat of distinctly different materials from the core (Figure 1).

Polymeric MPs mainly comprise polymers which determine their structure and significantly affect their properties. Ideally, polymers should be inert, stable, safe, biodegradable, biocompatible, and low cost.^3^ A wide range of polymers is used to prepare MPs derived from numerous natural and synthetic sources. Table 1 shows examples of different types of polymers.^4-6^

**Table 1:** Types of polymers used in microparticle formulations^4-6^

**Natural Polymers**			**Synthetic Polymers**	
**Proteins**	**Polysaccharides**	**Waxes**	**Biodegradable**	**Non-biodegradable**
Albumin	Starch	Beeswax	Carboxymethyl cellulose sodium	Fumaryl diketopiperazine
Gelatine	Chitosan	Carnauba wax Paraffin	Methylcellulose	Polyethene glycols
Casein	Sodium alginate		Hydroxypropyl cellulose	Poly-(N-isopropyl acrylamide)
Whey protein	Poly dextran		Hydroxypropyl methylcellulose	Acrolein
Soy protein	Pectin		Ethylcellulose	Epoxy polymers
Gluten	Sodium hyaluronate		Cellulose acetate butyrate	
Zein	Guar gum		Poly (lactic acid)	
	Konjac gum		Polylactic acid-glycolic acid copolymer	
	Carrageenans		Polyacrylic acid (Carbopol)	
	Agarose		Polymethacrylates	
	Tragacanth			
	Gum Arabic			
	Gellan gum			
	Xanthan gum			

The drug released from the MPs can be modulated depending on the nature of the polymer. Loading drugs at higher concentrations into the MPs, i.e., high entrapment efficiency, can be optimized based on the polymer type.^7^ In addition, studies proved that particle size directly affects the drug loading capacity, where reduction in particle size leads to a reduction in drug loading capacity and vice versa.^8^

Commonly, the surface morphology of the MPs originates from the chemical nature of the particle and the method of MP fabrication. It can be detected by different means, such as scanning electron microscopy. The surface morphology influences the properties of MP, such as wettability and adhesiveness.^9^ It was reported that the wettability of MPs is improved upon a higher number of surface asperities and roughness. On the other hand, the surface roughness was found to have an inverse effect on the adhesion of particle.^10^ As the surface roughness increases, the pull-off force is significantly reduced, thereby decreasing the adherence properties of the MPs.^11^

Another crucial aspect that should be considered during MPs preparation and characterization is electric charge of particles. Zeta potential is the standard analytical method of surface charge determination in a colloidal system. It can be used to determine the long- and short-term stability of the microparticulate colloidal dispersion. The colloidal system with high zeta potential (negative or positive) is regarded as an electrically stable system owing to the repulsive forces between particles. Low zeta potentials systems are at risk of coagulation or flocculation, possibly leading to poor physical stability.^12^

Microparticulate drug delivery system (MDDS) attracts attention due to its wide range of beneficial technological characteristics. Compared with the conventional dosage forms, MDDS offers numerous advantages, such as ensuring controlled and prolonged drug release pattern,^7^ reducing drugs dose and toxicity, improving drugs bioavailability, and enhancing the solubility of poorly soluble drugs due to their very wide surface area. Furthermore, they protect the drug from the in vitro/in vivosurrounding environment, target the drug to a specific biological site of action, mask the unsuitable taste and odour, and reduce dosing frequency, thus improving patient compliance.^13-19^ However, MDDS must be safe for successful clinical applications, perform therapeutic functions, provide comfortable administration routes, and be easily manufactured. The production of the MDDS showed some limitations due to its low reproducibility, costly materials, and manufacturing procedure, as well as some of their components and excipients that degrade into hazardous materials, which could be harmful to the environment.^2^ However, many novel microparticulate products are currently in clinical trials; however, some have been made available on the market. Table 2 demonstrates examples of commercially marketed products containing MPs.^20-30^

**Table 2:** Commercially marketed products formulated using microparticles

**Trade name**	**Generic name**	**Pharmaceutical company**	**Indication**	**Particle type**	**Reference**
Micro-K® Extencaps®	Potassium chloride extended-release	Ther- R.X. corporation	Hypokalemia	Microcapsules	^20^
Cotazym®	Pancrelipase	Organon	Pancreatic insufficiency	Microcapsules	^21^
Lupron Depot®	leuprolide acetate	Abbott Laboratories	Management of endometriosis	Microspheres	^22^
Nutropin Depot®	Somatropin (rDNA origin)	Genentech, Inc	Hormone deficiency	Microparticles	^23^
Sandostatin® LAR	Octreotide acetate	Novartis	Severe diarrhoea and flushing episodes associated with metastatic carcinoid tumors	Microparticles	^24^
Trelstar®	Triptorelin pamoate	Actavis Specialty Pharmaceuticals Co.	Palliative treatment of advanced prostate cancer	Microgranules	^25^
Vivitrol®	Naltrexone	Alkermes, Inc.	Prevention of relapse to opioid dependence	Microspheres	^26^
Decapeptyl*®*	Triptorelin pamoate	Ferring Pharmaceuticals	Metastatic prostate cancer	Microparticles	^27^
Risperdal Consta®	Risperidone	Vetter Pharma Fertigung GmbH & Co. KG.	Antipsychotic	Microspheres	^28^
Bydureon®	Exenatide	AstraZeneca Pharmaceuticals L.P.	Type 2 diabetes mellitus	Microspheres	^29^
Signifor® LAR	Pasireotide	Recordati Rare Diseases, Inc.	Acromegaly	Microspheres	^30^

Diabetes mellitus (DM) has emerged as a global health problem in the past few decades and has been declared the fifth leading reason for mortality in most countries,^31^ as DM is deemed a fundamental risk factor for cardiovascular diseases and renal problems.^32^ Basically, DM is a chronic hyperglycemic metabolic disorder that occurs due to multiple causes and is characterized by the improper metabolism of fats, carbohydrates, and protein.^33^ There are two main types of DM: type 1 and type 2. Absolute deficiency of insulin is the primary cause of type 1 DM. In contrast, impaired insulin secretion, insulin resistance, and increased glucose production are the causes of type 2 DM. Therefore, both DM types can be treated with insulin. However, hypoglycemic drugs can be used to manage type 2 DM.^34^

Despite the numerous antidiabetic medications that are flooding into the pharmaceutical market, a complete cure of DM remains unattained mainly due to the serious adverse effects of these drugs, such as hypoglycemia, gastric irritation, nausea, diarrhoea, and injection phobia, among others.^35^ Eventually, these drugs will result in poor patient compliance and low adherence to treatment. Therefore, designing a stable and non-invasive drug delivery along with controlled-release could be more therapeutically effective.^36^

Most significantly, the literature reported that microparticulate formulations could be promising to maintain a controlled blood concentration of the antidiabetic medications,^37^ improve the dissolution and release of drug,^38^ and ultimately, enhance their pharmacokinetics and bioavailability.^39^ Furthermore, surface modified and mucoadhesive MPs showed advantages in a protective effect against enzymatic degradationand enhancing peptide stability^40^ in addition to site-specific drug delivery^36^ and gastric retaining.^41^

 The literature revealed that the field of drug delivery has moved at an unprecedented pace, and a variety of drug delivery systems have taken centre stage over the past decade. Therefore, this review includes an inclusive outline of MDDS and focuses on their therapeutic applications as efficacious carriers for antidiabetic drugs and illustrates the global trend of research conducted in this area.

## Fabrication techniques of MDDS

###  Single emulsion technique

**Figure 2:**
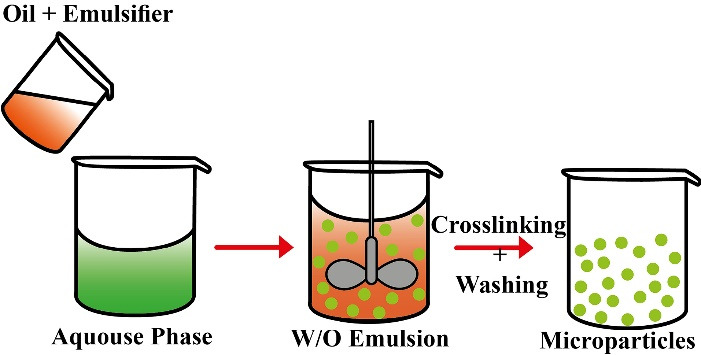
Single emulsion technique for the preparation of microparticles.

This method is used to prepare natural polymers-based MPs as proteins and carbohydrates. First, the polymer is dissolved in the aqueous medium, followed by its dispersion in a non-aqueous solvent as oil. Then, crosslinking of the dispersion is performed either by heating or using chemical crosslinkers as glutaraldehyde (Figure 2). The type of surfactant favorably influences the particle size, particle charge, surface morphology, drug loading, drug release, and bio-performance of the MPs.^42^

###  Double emulsion technique

**Figure 3:**
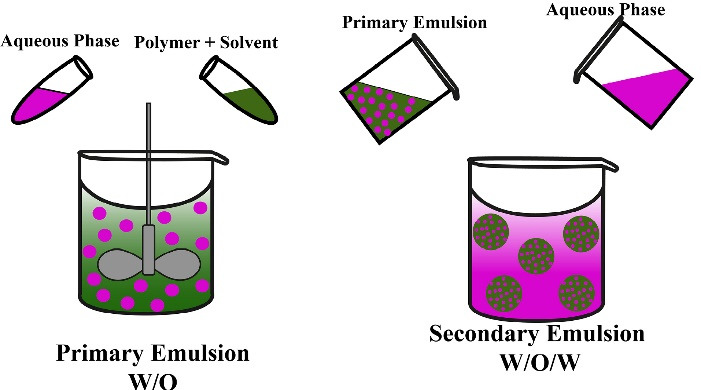
Double emulsion technique for the preparation of microparticles

Double emulsion technique comprises the formulation of double emulsions water-in-oil-in-water (w/o/w) or oil-in-water-in-oil (o/w/o). Both natural and synthetic polymers can be incorporated to prepare MPs. The double emulsion w/o/w (Figure 3) is more suitable for water-soluble drugs, peptides, proteins, and vaccines. For example, a luteinizing hormone-releasing hormone (LH-RH) agonist was successfully encapsulated into the MPs using the double emulsion method.^43^

###  Spray drying technique

**Figure 4:**
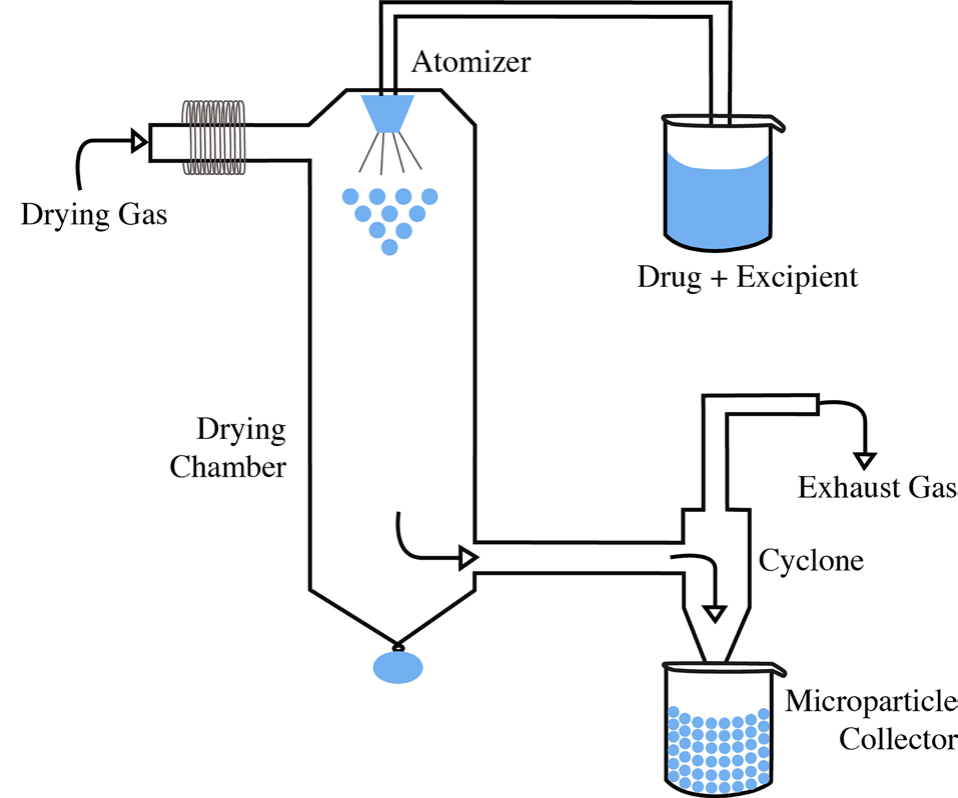
Spray drying technique for the preparation of microparticles

Both the polymer and the drug are dissolved in a volatile organic solvent and homogenized in a high-speed homogenizer (Figure 4). Subsequently, the resulting dispersion is sprayed in a hot air stream, where the solvent evaporates instantaneously and the MPs are formed.^44^

###  Solvent extraction

**Figure 5:**
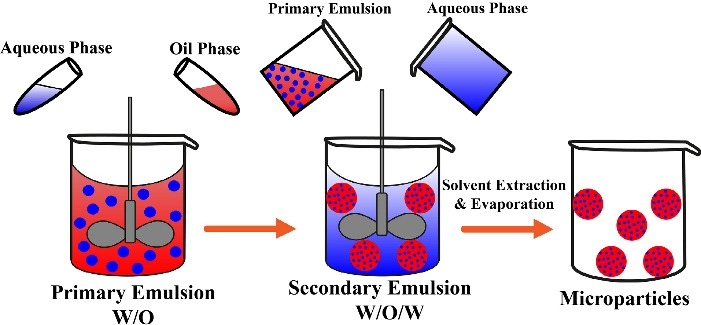
Solvent extraction technique for the preparation of microparticles

The solvent extraction or evaporation method is performed by dissolving the drug and the polymer in a suitable organic solvent. The mixture is then dispersed in an aqueous surfactant solution with stirring to form an emulsion.^45^ Finally, the MPs are collected after solvent evaporation (Figure 5). The main advantages of this method is the shorter hardening time and direct incorporation of the drug into the MPs.

###  Phase separation coacervation technique

**Figure 6:**
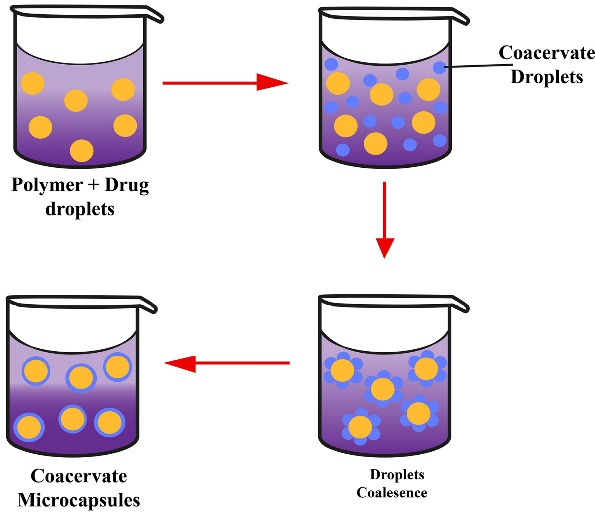
Phase-separation coacervation technique for the preparation of microparticles

This technique principally prepares the reservoir systems to encapsulate hydrophilic drugs, such as peptides and proteins. Its principle relies on the reduced polymer solubility in the organic phase to form a polymer-rich phase called a coacervate. Then, a third component is added to the system to separate the coacervate, forming two phases: supernatant and polymer-rich phases (Figure 6). In addition, phase separation can be achieved by different techniques such as salt, non-solvent, or incompatible polymer addition.^46^

## Factors affecting drug release from MDDS

 Drug release from MPs is affected by several factors, including:

###  Drug content

The drug release rate is affected by the amount of drug present in the MP, where the release increases with increasing drug concentration in the MP.^47^

###  Drug physical state

The physical state, molecular dispersion/crystalline structures of a drug affect the drug release kinetics from the MPs.^48^

###  Molecular weight of polymer

The molecular weight of polymer affects its erosion, where the molecular weight is inversely proportional to the release rate. Therefore, as the molecular weight increases, the diffusivity decreases, thus resulting in a lower drug release rate. In addition, many drugs are released by diffusion through water-filled pores, where the polymer degrades to form soluble monomers and oligomers. Hence, there is faster development of these tiny products, with the polymers having a lower molecular weight.^49^

###  Copolymer concentration

The co-monomer ratio in copolymers affects the drug release rate; when a more rapidly degrading monomer is used in the polymer, the release rate increases. Likewise, the release rate depends on the polymer erosion, in which the use of smaller and more soluble monomers will result in increased release rate.^50^ Nevertheless, the copolymer composition may be influenced by difference in the phase behavior of polymer or the thermodynamics of encapsulated active ingredient. For example, Zhang et al prepared poly(lactide-co-glycolide) (PLGA)-based MPs for the oral delivery of a poorly water-soluble drug, progesterone, to improve its physiological dissolution and bioavailability. It was found that the in vitro drug release was directly influenced by the copolymer composition; hence, the reduction of lactide content of PLGA was able to achieve further drug release.^51^

###  Types of excipients

Excipients have various crucial functions in the formulation; for example, they may influence the release of a drug through various mechanisms and its encapsulation effectiveness. Yanget al improved the encapsulation and uniformity of size distribution of bovine serum albumin (BSA) in MPs by including polyvinyl alcohol (PVA) in the formula. The increased PVA concentration increased the porosity of the MPs and controlled the release of BSA.^52^ Jain et al prepared myoglobin MPs-containing mannitol as a stabilizer. Their results showed that the addition of mannitol had improved the release rate of myoglobin by increasing the initial porosity of the MP’s matrix, leading to faster formation of the pore network within the sphere.^53^

###  Nature of the polymer 

The type of polymer used in MPs formulation and the functional groups that affect polymer degradation significantly affect its release rate. Polymers are categorized into two types: surface eroding and bulk-eroding. For bulk-eroding polymers, such as PLGA, this type of polymer allows rapid water permeation into the MP matrix, thus causing polymer degradation and a drug burst, where 50% of the drug is released during the first hour of the run, followed by a controlled release.^54^ Meanwhile, the surface-eroding polymers as polyanhydrides are made of hydrophobic monomers linked by labile bonds. It resists water penetration and degrades into oligomers and monomers at the polymer/water interface via hydrolysis. The drug release occurs at the surface as the polymer degrades.^55^

###  MPs size

Generally, the size of MPs impacts the loading capacity of the drug into the MPs and the drug release profile.^4^ As the particle size decreases, the ratio of their surface area to volume increases, and thus, the diffusion of drug particles and the release rate increases. On the other hand, the small-sized of the MPs results in a higher penetration of water into the particles and their decomposition, causing an immediate burst release of their content rather than the continuous release of the drug from the particle surface.^56^

###  Environmental pH

Some pilot studies have shown that the pH of the medium significantly affects the degree of hydration and swelling of crosslinked hydrophilic polymers.^57^ The swelling of the polymer having acidic or basic functional groups depends upon the pH of the surrounding medium relative to the corresponding p*K*_a_ and p*K*_b_ values of the functional groups. For instance, in the anionic polymer (e.g. having carboxylic, –COOH functional groups), the ionization of the acidic functional groups results in the production of negative charges on the surface of the polymer that can interact with the opposite positive charges in the medium. In addition, polymer erosion is also affected by the environmental pH. Hence, the swelling and or degradation of the pH-sensitive polymer that controls the drug-release profile from the MPs are affected.^58^

Moreover, the degree of ionization of the functional groups on the surface of the polymer and the surface of the mucous membrane, is also influenced by the hydrogen ion concentration in the surrounding medium. Therefore, the time and degree of contact between MPs-including mucoadhesive polymers and the absorption site are influenced.^59^ Researchers proved that positively charged polymers, such as chitosan,^60^ showed better mucosal adhesive properties than anionic polymers, providing favorable drug release and absorption conditions.

###  Route of administration 

Several formulation concepts are used to manufacture MDDS through different routes of administration, such as the transdermal, oral, ophthalmic, vaginal, and pulmonary for drug inhalation. Each of these routes of administration is characterized by certain physiological factors, such as tissue structure, pH of the medium, permeability barriers, and metabolic enzymes, all of which ultimately govern the release pattern and mechanism of the drug from the microparticulate carriers.^61^

## Therapeutic applications of antidiabetic loaded microparticles

 Since its discovery, MDDS has been extensively investigated and successfully used to encapsulate water-insoluble and water-soluble drugs. In addition, antidiabetic drugs, including insulin, were incorporated into MDDS due to their beneficial properties such as improving therapeutic efficacy, gastroretentive drug release, targeting medicinal compounds, improving insulin stability, reducing side effects, enhancing drugs dissolution, and attaining patient compliance.

###  Controlled gastroretentive drug release

One of the main methods of improving drug bioavailability is to retain the formulation in the stomach for a long duration.^62^ Various antidiabetics-loaded gastroretentive drug delivery systems had been proposed and evaluated (Table 3), including the floating and mucoadhesive MPs. The buoyancy of the MPs can be achieved when their bulk density is less than that of the gastric fluid.

**Table 3:** Various gastroretentive delivery systems for antidiabetic medications

**Type of System**	**Antidiabetic API**	**References**
Floating microspheres/microparticles	Repaglinide	^41^
	Rosiglitazone maleate	^63^
	Gliclazide	^64^
	Sitagliptin	^65^
	Metformin HCl	^66,67^
Effervescent floating tablets	Pioglitazone HCl	^68,69^
	Metformin HCl	^70,71^
	Rosiglitazone HCl	^72^
Non-effervescent floating tablets	Linagliptin	^73^
Microsponges/microballons	Mitiglinide calcium	^74^
	Metformin HCl	^75,76^
	Rosiglitazone maleate	^77^
Mucoadhesive matrix tablets	Metformin HCl	^78^
	Rosiglitazone maleate	^79^
	Repaglinide	^80^
Mucoadhesive microspheres	Linagliptin	^81^
	Glipizide	^82^
In situ hydrogel/superporous hydrogel	Mitiglinide calcium	^83^
	Rosiglitazone maleate	^84^

Another approach is preparing hollow MPs, so the formula stays buoyant in the stomach without affecting gastric emptying time and rate. MPs release the drug faster by floating on the gastric content resulting in an increased gastric residence time and controlled plasma concentration.^85^ In addition, the distinctive advantages of floating MPs reduce dosage frequencies, and possibility of mucosal adhesion and dose dumping.^86^

Dubey et al prepared the floating microspheres to retain metformin in the stomach and continuously release the drug in a controlled manner up to a predetermined time.^87^ Other studies followed the same technique to improve repaglinide’s bioavailability and efficacy.^88^ Furthermore, Shams et al loaded repaglinide successfully into floating microspheres prepared from different viscosity grades of hydroxypropyl methylcellulose (HPMC) polymer. It was predicted that the prepared repaglinide-loaded floating microscopic globules can provide a novel choice for a safe, economic, and increasingly bioavailable formulation to treat diabetes effectively.^89^

On the other hand, metformin hydrochloride was loaded to gastric-mucoadhesive MPs for sustained gastric residence time. Carbopol-934P/ethyl cellulose polymers as mucoadhesive MPs were prepared via the emulsification solvent evaporation technique. Results proved that incorporating metformin into the MPs would increase the drug bioavailability and improve glucose control in diabetic patients.^71^

###  Improving drug dissolution

Oral bioavailability depends on several factors, including aqueous solubility and dissolution rate. Further studies suggested MPs to enhance the solubility and dissolution rate of the lipophilic hypoglycemic drugs. For example, conventional glibenclamide tablets’ low oral bioavailability necessitated a novel formulation, MPs, to improve its low water solubility.^90^ Siafaka et al also developed polymeric MPs for oral delivery of glibenclamide using two biocompatible polymers, poly(e-caprolactone) and poly(butylene adipate). The in vitro drug release from the MPs was higher than that of the pure drug and in a sustained pattern ideal for reducing the daily dose of glibenclamide.^14^ Tzankov et al investigated the mesoporous silica MPs to improve glimepiride solubility and dissolution rate. The newly developed MPs were investigated in vitro and in vivo and found to possess high loading capacity and safe promising carriers to enhance the solubility of poorly soluble hypoglycemic drugs.^91^

Moreover, pioglitazone solubility and dissolution were improved by its incorporation into hydrophilic MPs. The MPs were prepared by spray-drying technique from two water-soluble components, poloxamer 407 and β-cyclodextrin. The spray-dried particles significantly increased the percentage of drug release rate compared to the control, pure pioglitazone.^92^

###  Reducing side effects

Generally, drug side effects are a fundamental obstacle in the development of therapeutic agents. Among various modified drug delivery systems, polymeric MPs are employed to enhance drugs safety and therapeutic activity. For the past few decades, MDDS had been extensively studied on its ability to deliver drug molecules to the target site of action to minimize undesired harmful effects and improve patients’ safety and compliance.^93^ For example, Volpatti et al were able to produce glucose-responsive insulin delivery systems in an injectable formulation for blood glucose control and hypoglycemic avoidance. The glucose-responsive delivery system was developed by encapsulating glucose-responsive, acetylated-dextran MPs in porous alginate microgels to improve glycemic control by releasing insulin into the blood, thereby detecting an elevation in the blood glucose levels.^94^

Moreover, catechin is a natural molecule that possesses antidiabetic activity, but a significant disadvantage of it is that it causes obesity. To overcome this problem, scientists have encapsulated catechin into Eudragit RS100 MPs. Results showed no signs of obesity in rat models even after 60 days of oral administration.^95^ Repaglinide requires frequent administration due to its short half-life, which may cause many adverse effects, as skeletal muscles pain, headache, and gastrointestinal (GI).^96^ Sharma et al encapsulated the drug into microspheres to modify the drug release, thereby controlling its concentration for a prolonged duration and reducing its side effects.^41^ Ethyl cellulose MPs containing metformin HCl were developed by emulsification solvent evaporation technique. The sustained release of the drug from these MPs was more prominent at a phosphate buffer of pH 6.8 than in the simulated gastric medium. Thus, the authors proposed ethyl cellulose MPs as a convenient carrier for water-soluble hypoglycemic drugs as metformin HCl in managing type 2 DM.^97^

###  Targeting drugs to the site of disease

####  Magnetic microparticles

The magnetic MPs were employed to localize the drug to the site of the disease. The freely circulating drug was targeted to the receptor site and maintained at the therapeutic concentration for a specific period. This mechanism was achieved by incorporating nano/micromagnets into the polymeric MPs, e.g., chitosan and dextran, and exposing them to an external magnetic field for their immobilization.^98^ There are two types of magnetic MPs: therapeutic magnetic MPs and diagnostic magnetic MPs, where the former one was used to deliver proteins, peptides, and chemotherapeutic agents to tumors as liver tumors^99^; and the latter was used for imaging liver metastases, distinguishing bowel loops from other abdominal structures by forming nano-sized particles super-magnetic iron oxides.^100^ The literature is full of studies that report magnetic particulate carriers for delivering antidiabetic medications to a localized disease site. In a previous study, the influence of polymer composition on insulin release was investigated by exposing ethynyl vinyl acetate MPs to the oscillating magnetic field.^101^ Insulin-magnetite- PLGA MPs were orally administered to mice in the presence of an external magnetic field. A significantly improved hypoglycemic effect (blood glucose levels reduced to 43.8%) was observed, indicating the efficacy of the magnetic microspheres in oral insulin therapeutics.^102^

Moreover, Teply et al prepared the negatively charged insulin-loaded PLGA MPs-complexed with positively charged micromagnets. The complexes were effectively localized in a mouse small intestine in vitro model by an external magnetic field application, indicating that the complexes encapsulating insulin (120 units/kg) were stable. They exhibited long-term blood glucose reduction in the mice groups fitted with magnetic belts and significantly improved insulin bioavailability compared to the control.^103^ Alginate-chitosan beads containing magnetite nanoparticles were placed as a system to control insulin release in the presence of an oscillating magnetic field. Beads entrapment efficiency was 35%, and the magnetic field increased three times in the insulin release.^104^

####  pH-Sensitive microparticles

Situ et al prepared insulin-loaded oral bioadhesive MPs coated with a resistant starch-based film to deliver antidiabetic bioactive drugs to the colon. The starch was chemically modified to enhance its stability and resistibility to GI enzymatic degradation. Results proved the MPs’ effectiveness to control the average plasma glucose levels up to 22 hours in diabetic rats. Then further development for the resistant starch-based coat was conducted through its conjugation with concanavalin A glycoprotein. The modified coat showed better colon targeting and maintained the hypoglycemic effect of insulin for 44–52 hours in diabetic rats.^105^

Another approach for colon targeting was attempted by preparing sodium alginate MPs containing the bile salts as permeation enhancers. Gliclazide was loaded into sodium alginate MPs containing chenodeoxycholic acid^106^ and deoxycholic acid^107^ bile salts. The formulations showed extended gliclazide in vitro release profiles and successful colon targeting properties. Leong et al developed pH-responsive carboxymethylated *kappa*-carrageenan MPs to protect insulin from GI degradation. The prepared formula was further surface-lectin-functionalized to enhance the intestinal mucoadhesion. The surface-modified formulation demonstrated accurate colon targeting and could maintain the hypoglycemic effect for up to 24 hours in diabetic rats.^108^ Chitosan-snail mucin MPs were prepared for pH-sensitive oral delivery of insulin. In vitro release profile of insulin was evaluated in two pH environments (pH 1.2 and pH 7.4) in animal models. Results showed retarded release in the acidic medium; however, the continuous release of the alkaline medium was prolonged for up to 12 hours. Animal models controlled the normal average blood glucose levels for up to 8 hours.^40^

###  Improving insulin stability

Insulin instability in the gut limits its administration to the parenteral route only. Several approaches had been proposed to overcome this problem to improve oral insulin stability and bioavailability. For instance, Sajeesh et al complexed methyl-β-cyclodextrin to polymethacrylic acid hydrogel MPs to be tested for oral insulin delivery in diabetic animal models. Cyclodextrin was responsible for stabilizing insulin by reducing its self-aggregation. Results also showed enhancement in insulin’s oral absorption.^109^ Carboxymethyl β-cyclodextrin grafted carboxymethyl chitosan hydrogel MPs were found promising for oral insulin administration.^110^ Another study was performed by encapsulating insulin into mucinated sodium alginate MPs. The MPs effectively lowered blood glucose levels in rabbit diabetic models after 5 hours of their oral administration. MPs surface modification is another technique that was tried for oral insulin delivery. Chitosan-snail mucin based microspheres were fabricated and loaded with insulin. The loading capacity was high, and the in vitro release was above 80% over 12 hours. The insulin-loaded MPs significantly reduced blood glucose levels in mice compared to the positive control, and the effect continued for 8 hours.^111^ Acryl-EZE enteric polymer-coated MPs containing surfactin and iturin lipopeptides could achieve only 7.67% of oral relative bioavailability of insulin. Nevertheless, these MPs maintained the postprandial blood glucose level of about 50% of the initial dose, similar to the subcutaneous injection.^112^ In addition, glucan MPs thickened with thermosensitive poloxamer 407 gels were suggested to be potential insulin oral carriers.^113^

The multilayer-coated MPs using a layer-by-layer polymers installation was another assessed approach. Balabushevich et al generated the layer-by-layer MPs from dextran sulfate and chitosan polymers.^114^ Meanwhile, in another study, the MPs were coated with alternating layers of poly(vinyl alcohol) and poly(acrylamide phenyl boronic acid-co-N–vinyl caprolactam) on the surface of PLGA MPs.^115^ Shrestha et al prepared the annealed thermally hydrocarbonized porous silicon (AnnTHCPSi) and undecylenic acid-modified AnnTHCPSi (AnnUnTHCPSi) MPs for oral insulin delivery. The surface of MPs was modified using chitosan to enhance insulin’s intestinal permeation. Insulin intestinal permeation was evaluated in Caco-2/HT-29 cell co-culture monolayers. The chitosan-coated MPs showed a significant improvement in insulin penetration through the cells.^116^

###  Natural products-loaded MPs

Gongronema latifolium is a conventional herbal medicine plant used to treat various diseases, including diabetes. The plant extract was loaded into the solid-lipid MPs with a retention efficiency of 68%. In addition, the mean percentage reduction in blood glucose after oral administration of the extract loaded MPs was 76% and 24.4% compared to the reference glibenclamide, which resulted in 82.6% and 46.7% at 2 and 12 hours, respectively.^117^

Catechin a natural molecule that possesses antidiabetic activity. Its low oral bioavailability limits its uses. However, catechin encapsulation into Eudragit RS100 MPs significantly improved its absorption and reduced blood glucose levels in diabetic rats. The blood glucose level of the catechin MP treated group was found to be (119.37 ± 12.46 mg/dL) after 60 days of treatment compared to (206.54 ± 9.54 mg/dL) of the hyperglycemic rats.^95^

Berberine active constituent is found in several plants as European barberry, goldenseal, Oregon grape, and tree turmeric. It has attracted much interest in recent years due to its potential as a natural alternative to other synthetic antidiabetic drugs. Unfortunately, the low oral bioavailability is limiting its development for further clinical treatments. Recently, researchers have attempted to improve its oral hypoglycemic effect by incorporating the berberine-phospholipid complex into the phytosomes delivery system.^118^ Some bioflavonoids, such as rosmarinic acid from the plant Lamiaceae, had been used as antidiabetic drugs and antioxidants. Rosmarinic acid crosslinked MPs contributed a more substantial inhibitory effect on α-glycosidase along with reduced cytotoxicity and antioxidant activity than the free compound.^119^

###  Sustained drug release

Various strategies were investigated for the extended-release formulations of antidiabetic drugs such as matrix sustained-release tablets, orodispersable tablets, and depots.^120-122^ Nevertheless, MDDS had granted great attention to this application. Biodegradable polymeric MPs were used extensively to retard drug release, reduce dosing frequency, and enhance bioavailability and safety.^123^ The biodegradable MPs are made from either natural polymers as starch or synthetic polymers, such as PLGA.^124^ Biodegradable polymeric MPs swell and form a gel-like structure when in contact with an aqueous medium at the mucous membrane. The rate and extent of the drug release are dependent on the polymer itself and its concentration. The main challenges in the formulation of biodegradable polymeric MPs are drug loading efficiency and drug release controlling.^125^

Synthetic polymeric MPs were used as drug delivery vehicles in clinical trials due to their safety and biocompatibility. However, they have some limitations, such as their migration tendency away from the injection site, leading to a potential risk of embolism and further organ damage.^126^ Wu et al prepared insulin-loaded porous microspheres to control blood glucose levels for at least 18 days. The carrier acquired a unique glucose sensitivity property due to incorporating glucose oxidase, where insulin was released from the delivery system upon elevated blood glucose levels.^115^ For example, exenatide, an antidiabetic drug with a short half-life, was loaded into porous MPs to improve its characteristics. It was reported that the prepared exenatide-loaded porous microspheres had a sustained release for 30 days in rat models.^127^

Furthermore, rosiglitazone maleate mucoadhesive microspheres were prepared for achieving controlled drug release. The mucoadhesive microsphere tended to adhere to the mucosal tissue for a prolonged period of 12 hours.^128^ In another study, polylactic acid MPs were approved as a successful sustained release delivery system of metformin hydrochloride in the treatment of diabetes. The MPs improved the drug bioavailability and overcame the difficulty of oral tablet swallowing, which could be considered a potential alternative to oral pills.^129^

###  Antidiabetic drugs mucosal delivery 

**Figure 7:**
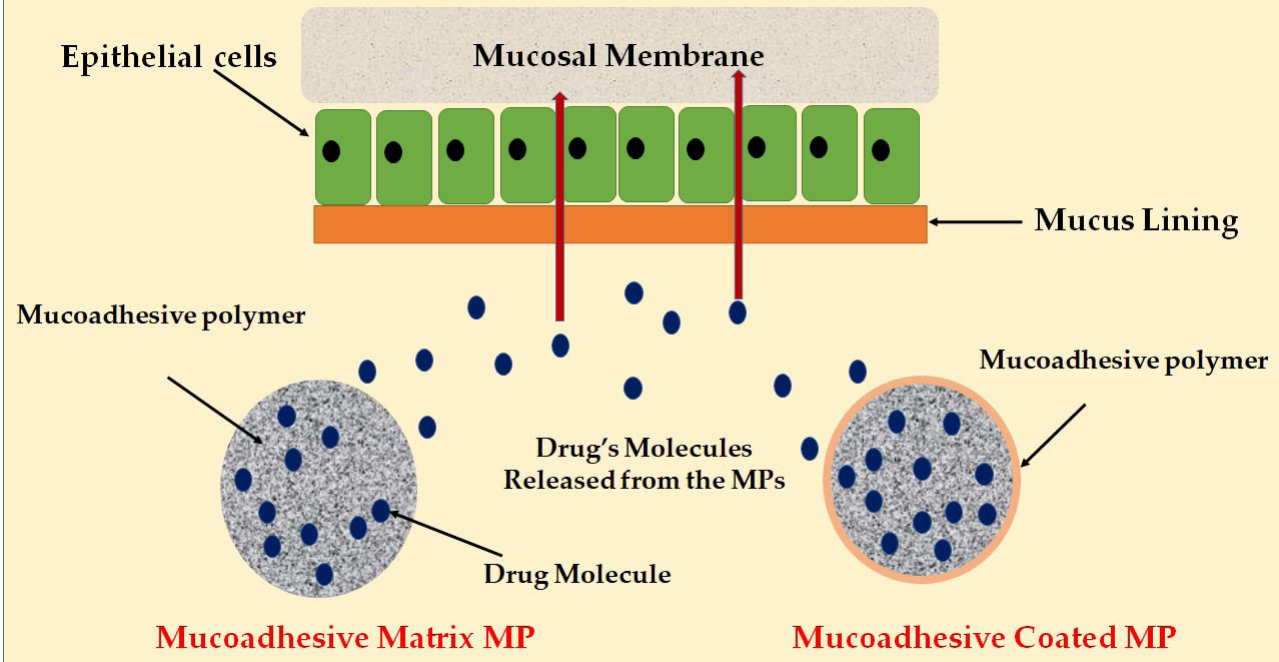
A scheme demonstrates the release of drug’s molecules from the mucoadhesive microparticles at the site of absorption

Adhesion describes the sticking and bioadhesion as sticking a drug to the membrane using water-soluble polymers. The mucoadhesive MPs are intrinsically prepared by incorporating a mucoadhesive polymer-based matrix in the formulation or coating the MPs with a mucoadhesive polymer.^130^ These MPs have offered several advantages over the conventional formulations, including a prolonged residence time at the application site, controlled drug release, and enhanced drugs permeation and bioavailability.^131^ Mucoadhesive MPs may be delivered to different body sites lined with mucous membrane, such as the oral, buccal, ocular, rectal, vaginal, and nasal. Figure 7 displays the mechanism of the drug release from the MPs at the target site of absorption.

However, the mucoadhesive efficacy of a dosage form is dependent on various factors, such as the nature of the mucosal tissue and the physicochemical properties of the polymeric formulation. These mucoadhesive agents are typically high molecular weight polymers of high molecular weight and interact with the mucus layer of the mucosa epithelium through hydrogen bonding, ionic, hydrophobic or van der Waals interactions.^132^ Table 4 describes several examples of the mucoadhesive MPs on their polymer components, preparation method, administration routes, and aim of the preparation.

**Table 4:** Examples of antidiabetic drugs-loaded mucoadhesive microparticles

**API**	**Delivery system**	**Polymer(s)**	**Method of preparation**	**Route of administration**	**Aim**	**Reference**
Insulin	Hydrogel microparticles	Whey protein/ alginate	Cold gelation technique and an adsorption	Oral	Improvement of intestinal absorption and drug bioavailability	^131^
Metformin	Gastroretentive discs		Emulsification, solvent evaporation, and compression	Oral	Gastroprotective formulation to improve therapeutic performance	^132^
Insulin	Microsphere	CP/EC	Spray drying	Nasal	Non-injectable system for insulin	^133^
Insulin	Polyelectrolyte microparticles	Fumaryl diketopiperazine	Aggregation	Oral	Improve bioavailability	^134^
Insulin	Hydrogel microparticles	Chitosan and Dextran sulfate	Ionic gelation	Oral	Improve the oral delivery of proteins/peptides	^135^
Insulin	Microsphere		Spray drying	Nasal	Improve the systemic absorption	^136^
Insulin	Multicomponent microparticles	PMAA/PEG/chitosan	Layer-by-layer assembly	Oral	Improve bioavailability	^114^
Metformin HCl	Microparticle compressed into discs	Chitosan/PVA	Emulsification solvent evaporation	Oral	Controlled drug release and enhancing bioavailability	^88^
Insulin	Microspheres		Membrane emulsification	Oral	Improve bioavailability and oral delivery	^137^
Exenatide	Microparticles	Dextran sulfate/Chitosan	Coprecipitation and Micronization	Nasal	Enhanced drugs permeation	^138^
Sitagliptin	Microsphere	Carbomer 934P/E.C.	Spray drying	Oral	Controlled drug release	^139^
Metformin	Microsphere		Ionic gelation	Oral	Sustained release and enhance absorption	^140^
Repaglinide	Microparticle	Chitosan	Spray drying	Nasal	An alternative route of administration	^141^
Glipizide	Microbeads	PAA	Emulsification solvent evaporation	Oral	Prolonged-drug release	^142^

CP: Carbopol 934; E.C.: Ethylcellulose; PMAA: Polymethacrylic acid; PEG: Polyethylene glycol; PVA: Polyvinyl alcohol; PAA: Polyacrylic acid.

## Routes of administration

**Figure 8:**
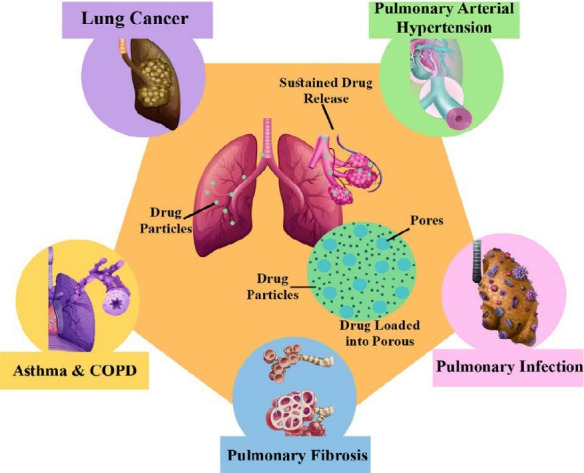
Pulmonary therapeutic applications and drug targeting of microparticles delivery systems

Various options for the administration of antidiabetics were proposed to treat patients with DM. As a result, researchers formulated the antidiabetic drugs-loaded MPs to be delivered in several administration routes for more appropriateness in patients’ perception. For example, the pulmonary route of administration^143^ had been used for many decades to deliver drugs for systemic and local applications to treat various respiratory system diseases (Figure 8). Recently, inhaled antidiabetic drugs-loaded MPs have become a highly focused research trend in the pharmaceutical industry. For instance, Rashid et al conducted a study evaluating rosiglitazone-loaded porous microspheres for pulmonary administration. The candidate inhaled formula was non-invasive and successfully released 87% of rosiglitazone within 24 hours.^19^

It is well-known that one of the key factors deciding the drug bioavailability is the residence time at the absorption site. The strategy of using mucoadhesive polymers was employed for this purpose. In a previous study, N-trimethyl chitosan MPs with permeation enhancers were prepared for pulmonary insulin delivery in diabetic rats. Chitosan-based MPs were found pharmacologically efficient and relatively bioavailable compared to subcutaneous administration. The histological examination of the rat’s lung proved the safety of the formula.^144^ Hamishehkar et al prepared long‐acting, respirable, biodegradable microcapsules loaded with insulin. The dry powder inhaler formulation was tested on diabetic rats through the pulmonary route. Results showed that the prepared microcapsules had a longer residence time and ability to control blood glucose levels for up to 48 hours.^123^

Buccal drug administration was another proposed route for the delivery of the antidiabetic drugs-loaded MPs. The bioadhesive metformin loaded MPs for the oromucosal administration was prepared by Sander et al The prepared formula was tested for its bioadhesive properties using an ex vivo flow retention model. The results indicated improved metformin retention on porcine mucosa, making it the right candidate for the buccal route of administration.^145^ Nasal and oral mucoadhesive MPs were the most commonly investigated delivery systems for insulin and oral hypoglycemic drugs. For example, the nasal delivery of insulin-loaded gelatin MPs was investigated in healthy rats. The hypoglycemic effect was assessed for both suspension and dry powder MPs formulations. A significant decrease in blood glucose levels was observed after the dry powder administration. The bioavailability enhancing effect of the mucoadhesive gelatin microspheres was attributed to the long residence time of the MPs at the nasal mucosa besides opening the tight intercellular junctions.^146^

Multilayered surface-modified MDDS were suggested for oral insulin delivery. The MPs were prepared via the alternative deposition layer by layer of ferric ions and dextran sulfate at the surface of the microspheres. Insulin hypoglycemic effect was mainly determined by the number of layers and lasted for 12 hours, with ten bilayers deposition.^147^ Table 5 displays examples of antidiabetic drugs-loaded polymeric MPs, their component polymer, preparation method, and administration route.

**Table 5:** Examples of antidiabetic drugs-loaded polymeric microparticles, component polymer, preparation method, and routes of administration

**Drug Name**	**Pharmacological class**	**Polymer(s)**	**Administration Route**	**Preparation technique **	**Reference**
Liraglutide	GLP-1 analogue	PLGA	Parenteral	W/O/W double emulsion	^148^
Exenatide	GLP-1 analogue	PLGA	Parenteral	W/O/W double emulsion	^149^
Insulin	Intermediate-acting insulin	PEG-grafted chitosan	Intranasal	Ionotropic gelation	^150^
Insulin	Insulins	Sodium alginate/chitosan	Oral	Coacervation phase separation	^137^
Metformin	Biguanides	Sodium alginate/chitosan	Oral	Spray-drying	^151^
Pioglitazone HCl	Thiazolidinediones	Eudragit RS 100/Eudragit RL 100	Oral	Solvent evaporation	^152^
Glipizide	Sulfonylureas	Eudragit RS 100 -RL 100	Oral	Emulsion crosslinking	^153^
Glipizide	Sulfonylureas	Galactomannan gum	Oral	Emulsion crosslinking	^154^
Tolbutamide	Sulfonylureas	Alginate and xanthan gum	Oral	Orifice-ionic gelation and emulsification gelation	^155^
Gliclazide	Sulfonylureas	Tamarind seed polysaccharide/alginate	Oral	Ionotropic gelation	^156^
Insulin	Insulins	PLA	Oral	Emulsion-solvent evaporation	^157^
Insulin	Insulins	Chitosan/PVP	Intranasal	Spray drying	^136^
Insulin	Insulins	Sodium alginate	Oral	Spray drying	^158^
Insulin	Insulins	Quaternized chitosan	Oral	Emulsification and crosslinking	^159^

PLGA: Poly(lactic-co-glycolic acid); GLP-1: Glucagon-like peptide-1; PEG: Polyethylene glycol; PLA: Poly9lactic acid); PVP: Polyvinyl Alcohol; SPG: Shirasu-porous-glass; W/O/W: Water-in-Oil-in-Water.

## Current status and future developments

Despite the global evolution in developing innovative microparticulate systems for the delivery of antidiabetic drugs, there are still numerous challenges due to the wide variations in drug loading, particles characteristics, and manufacturing processes. Therefore, there are few antidiabetic MPs-based products currently available in the market. For example, Bydureon® is a sustained-release injection in a pre-filled pen containing exenatide, a glucagon-like peptide-1 receptor agonist, for subcutaneous administration. This depot is produced by AstraZeneca U.K. Limited using microspheres technology, where the drug particles are loaded into PLGA based microspheres. This medication helps to control blood glucose levels in type 2 diabetic patients.^24^

Furthermore, to reflect the current status and future developments in this field, relevant patents published on Google Patent.com were also reviewed. Table 6 shows examples of patents/innovations (from year 2000 onwards) of antidiabetic drugs-loaded MPs related to patent applications.

**Table 6:** Patents-innovations (2000 onward) related to antidiabetic drugs loaded microparticles

**Patent number**	**Title**	**API**	**References**
US20040234615A1	Oral insulin composition and methods of making and using thereof	Insulin	^160^
WO2005092301A1	Insulin highly respirable microparticles	Insulin	^161^
US6444226B1	Purification and stabilization of peptide and protein pharmaceutical agents	Insulin	^162^
US20100055194A1	Pharmaceutical formulations containing microparticles or nanoparticles of a delivery agent	Insulin	^163^
WO2013115746A1	A production method for (effervescent) pharmaceutical compositions comprising an alpha-glucosidase inhibitor (Miglitol) and metformin	Metformin/Miglitol	^164^
CN102085355A	Liraglutide long-acting microsphere injection and preparation method thereof	Liraglutide	^165^
WO2017107906A1	Exenatide microsphere preparation and preparation method thereof	Exenatide	^166^
CN101658496A	Exenatide release microsphere preparation, preparation method and application thereof	Exenatide	^167^
EP2814460B1	Glucose-responsive microgels for closed-loop insulin delivery	Insulin	^168^
WO2015166472A1	Extended-release liquid compositions of metformin	Metformin	^169^
CN106176623A	Metformin hydrochloride PLGA microsphere and its preparation method and application	Metformin	^170^
JP2004501188A	Controlled release formulation of insulin and method thereof	Insulin	^171^
US20120121707A1	Tolbutamide particle and preparing method thereof and method of reducing a blood glucose	Tolbutamide	^172^
WO2008062470A2	Stabilized controlled release dosage form of gliclazide	Gliclazide	^173^
WO2014128116A1	A production process for gliclazide formulations	Gliclazide	^174^

## Conclusion

 MDDS offer several merits over traditional pharmaceutical dosage forms, such as increasing efficacy, reducing toxicity, and improving patient compliance and comfort. Several methods are used for MPs preparations such as single emulsion, double emulsion, spray drying, solvent extraction, and phase separation coacervation technique. The content and physical state of the drug; polymer’s nature, molecular weight, and concentration; and type of excipients used are the main factors affecting the drug release profile from the MPs. Diabetes is considered a global disease; nevertheless, research and development in drug delivery and disease management are ongoing to improve drugs efficacy and safety. Antidiabetics-laden MPs were created for their unique applications in targeting drugs to a specific site in the body, improving drug dissolution, controlling drug release, reducing side effects, and enhancing bioavailability and stability. The interest in applying MDDS for the treatment of diabetes for administration via different routes is currently increasing. Through the combination of different strategies, the MPs can be effectively placed and used, particularly in cell sorting, diagnosis, genetics, and biological products.

## Ethical Issues

 Not applicable.

## Conflict of Interest

 The authors declare that there is no conflict of interest in this paper.
